# Optimizing Interhospital Transfers in Orthopedics and Trauma Surgery: Challenges, Insights, and Proposals for Standardized Care in Germany

**DOI:** 10.3390/clinpract14030063

**Published:** 2024-05-08

**Authors:** Jonas Roos, Soufian Ben Amar, Leonie Weinhold, Alberto Alfieri Zellner, Amadeo Touet, Christian Prangenberg, Thomas Loy, Martin Gathen

**Affiliations:** 1 Department of Orthopedics and Trauma Surgery, University Hospital of Bonn, 53127 Bonn, Germany; 2 Institute for Medical Biometrics, Informatics and Epidemiology, University Hospital of Bonn, 53127 Bonn, Germany

**Keywords:** interhospital transfers, orthopedic complications, patient flows, collective analysis, survey

## Abstract

Background: Germany’s high density of under-equipped hospitals and anticipated surge in orthopedic and trauma surgery-related diseases by 2030, combined with personnel shortages, are expected to increase patient transfers between hospitals, an issue that urgently needs standardized protocols. Despite some existing cooperative agreements, such as between joint-replacement centers or within the Trauma Network DGU^®^, these measures do not adequately address the full range of patient-transfer cases, including those due to a lack of specialization or staff shortages, resulting in delayed treatment and potential health risks. This study aims to dissect the intricacies of interhospital transfers in orthopedics and trauma surgery across Germany, focusing on understanding the underlying reasons for transfers, comparing the operational structures of small and large hospitals, and laying the groundwork for future standardized protocols to enhance patient care. Material and Methods: A cross-sectional study was conducted in the form of an online survey via SoSci Survey, which was directed at orthopedic surgeons and trauma surgeons working in hospitals in Germany. The 22-question survey gathered information on participants’ clinic roles, departmental details, transfer processes, frequent diagnoses, perceptions of transfer quality, and improvement areas. The survey was sent to orthopedic and trauma surgeons in Germany by the specialist society. The data were analyzed using descriptive and inferential statistics to ensure a comprehensive insight into interhospital transfer practices. Results: The study involved 152 participants from various hospital ranks and located in different hospital sizes and types across rural and urban areas. A significant difference was observed between the care structures of basic/regular care and central/maximum care hospitals, especially regarding the available facilities and specialties. These findings suggest improvements such as better patient documentation, increased digital communication, optimized patient distribution, and standardization of transfer requests, among others. Conclusions: This study highlights the urgent need for improved protocols and resource allocation to eliminate inequalities in transfers between hospitals in orthopedics and trauma surgery in Germany.

## 1. Introduction

In May 2022, a commission led by the German Minister of Health Prof. Dr. Karl Lauterbach was established to address necessary reforms in the hospital sector, including the re-planning of inpatient and outpatient care regulations [1,2]. Currently, Germany has a high density of hospitals, but they are generally too small and insufficiently equipped in terms of specialized personnel and modern technical equipment [3,4]. This situation leads to care-related transfers between hospitals and is expected to worsen further by 2030 due to the anticipated significant increase in the burden of orthopedic and trauma surgery-related diseases, combined with a lack of nursing staff and overall personnel scarcity [5,6,7].

The structure of hospital care had already changed with the introduction of “diagnosis-related groups” (DRG) in the early 2000s, resulting in increased economic pressure, particularly on cost-intensive structures [8]. In 2005, the cost coverage of the total cost for polytraumatized patients was only 88% and a 50.7% increase in interhospital patient transfers (IHTs) was observed in the Bielefeld area from 2001 to 2006 [9,10].

To address the care of traumatized patients, the German Society for Trauma Surgery (DGU) launched the Trauma Network Initiative in 2004, which led to the development of the Trauma Network DGU^®^ for the sustainable improvement of care for polytraumatized patients in Germany [11]. As a result, larger centers are now obligated to co-treat and provide follow-up care for these patients [12]. In orthopedic surgery, mandatory cooperation agreements exist between joint-replacement centers to manage severe joint-replacement revision situations and complications [13]. Both medical and non-medical factors, such as the time of transfers, the gender of the transferred individual, and age, are relevant in patient care [14]. Despite existing regulations for the care of severely injured patients and complex cases, there are no established guidelines for the transfer of patients who require care due to a lack of specialization, the complexity of the disease, staff shortages, or other individual factors. Possible examples are patients showing a spondylodiscitis or complications after trauma surgery. Those cases often need fast and targeted care. In a recently conducted monocentric study, it was shown that these IHTs usually occur at short notice, and there are no structured protocols for these. Essentially, it is up to the attending physician of the sending department to determine whether the capacities and the expertise are sufficient to continue treating the patient or whether they need to be transferred. For the transfer, multiple hospitals must then be contacted to facilitate this. This takes time and delays further treatment. So far, there is no standardized protocol for these transfers [15].

Recent international studies on IHTs highlight that transfers outside of regular working hours lead to poorer patient outcomes and point to the critical gap in the organized structures for the management of transfers of critically ill patients [16,17]. In addition to the transfer process and the exchange of information, the actual transportation of patients is cited as a problematic area [18]. Concerns about the appropriateness of referrals are also notable, with a significant number of referrals considered inappropriate, indicating the need for clearer policies and better organizational procedures [19,20]. These findings call for improved frameworks to ensure more effective and appropriate patient transfers to improve overall patient care and outcomes.

The aim of this study was to understand the reasons for interhospital transfers and to capture the current situation in orthopedics and trauma surgery in Germany in order to improve the process. The objective was to identify how transfers take place, what the current problems with transfers are, which patients are transferred, and what the participants see as opportunities for optimization. In particular, the situation of smaller hospitals of basic/regular care (BRC) with 200–499 beds is compared with large hospitals of central/maximum care (CMC) with 500 to 1000 beds to better understand differences in the current care situation and problems with interhospital patient transfers. Based on this work, the foundations should be laid to improve the current transfer situation and to create structured protocols in the future to improve patient transfers in orthopedics and trauma surgery.

## 2. Materials and Methods

### 2.1. Study Design

In this study, we conducted a survey on interhospital transfers in the field of orthopedics and trauma surgery in Germany. A cross-sectional study was conducted in the form of an online survey via SoSci Survey (SoSci Survey GmbH, Munich, Germany), which was directed at orthopedic surgeons and trauma surgeons working in hospitals in Germany [21]. The questionnaire was available to participants from 15 March 2023 to 15 June 2023. All participants received a link to the questionnaire, through which they could answer questions as single-choice, multiple-choice, and open-ended response options. The questionnaire consisted of a total of 22 questions and took 5 to 10 min to complete. The questionnaire with possible answers has been made available under Supplementary Materials.

### 2.2. Participants

The participants were orthopedic surgeons and trauma surgeons working in Germany at various age and career levels. The participation in the study was voluntary, and the participants received no compensation. The questionnaire was distributed via the professional association and sent to the participants.

### 2.3. Questionnaire

The survey consisted of a total of 22 questions. These initially asked about the participant’s role in the clinic, the size and location of the clinic and department, and the size of the intensive care department. Further questions addressed the department’s specialization, certifications, and available imaging capabilities. Additional questions explored communication and the process of transfers, transfer time, duration of transfers, and reasons for transfers. The final section primarily involved the most frequent diagnoses for transfers, separated by orthopedics and trauma surgery. Participants were also asked about their current subjective quality of transfers, possibilities for optimization, and reasons for delays in patient transfers.

### 2.4. Inclusion and Exclusion Criteria

All participants who took part in the survey, and where the missing responses were ≤5%, were included. Questionnaires were excluded if the error rate was higher. There were no further exclusion criteria.

### 2.5. Ethics Approval

The study was approved by the local institutional review board (No. 157/21). This study was performed in accordance with relevant guidelines and regulations. Prior to the study, all participants provided their informed consent.

### 2.6. Declaration of Generative AI in the Writing Process

In drafting this manuscript, we used the services of GPT-4 to improve linguistic clarity and eliminate grammatical inconsistencies [22]. Rigorous manual checks were then performed, and the authors take full responsibility for the integrity of the content.

### 2.7. Statistical Analysis

Characteristics of the data are described using means with standard deviations (SD) or median [range] for continuous variables and frequency distributions with percentages for categorical variables. Percentages are calculated based on all available data, including any missing values.

Differences between hospitals of BRC and hospitals of CMC regarding patients’ characteristics or patient transfers were assessed by Fisher’s exact test for categorical variables and the Wilcoxon–Mann–Whitney U Test for continuous variables. Confidence intervals (CIs) for the median were generated by the percentile bootstrap method (1000 repetitions), and CIs for proportions were calculated using the Clopper–Pearson method. *p*-values were corrected for multiple testing by the Bonferroni method.

*p*-values < 0.05 were considered significant. All analyses were carried out using the R Software for Statistical Computing Version 4.3.0.

## 3. Results

### 3.1. Background Characteristics

A total of 152 participants took part in the study. Among the respondents, 20 (13.2%) were employed in a basic care hospital (200 to 299 beds), 36 (23.7%) in a regular care hospital (300 to 499 beds), 20 (13.2%) in a central care hospital (500 to 699 beds), and 74 (48.7%) in a maximum care hospital (700 to over 1000 beds); 2 (1.3%) did not disclose further details in this regard. Among these hospitals, 20.4% were local trauma centers (Level 3), 23.0% were regional trauma centers (Level 2), and 52.0% were supraregional trauma centers (Level 1). Five hospitals were dedicated orthopedic facilities, two were not trauma centers, and one was not yet classified. Table 1 shows an overview of the background characteristics of CMC and BRC participants.

### 3.2. Comparison between Basic/Regular Care and Central/Maximum Care

Dividing the participants’ hospitals into the groups of basic/regular care (200–499 beds) and central/maximum care (500 to 1000 beds) reveals significant differences in the care structures. In total, 56 (36.8%) participants were assigned to the BRC group, and 96 (63.1%) participants were assigned to the CMC group. Table 2 shows an overview of the statements from the CMC and BRC participants.

Clear differences were evident in the specializations of the two groups. Overall, there was a significantly higher rate of specializations and certifications in the CMC group (4 [1, 13] vs. 2 [1, 9] *p* < 0.001). Table 3 provides an overview of the specializations of the clinics.

### 3.3. Transfers

The participants were asked about their subjective impression of when the majority of patient transfers to other clinics take place. A total of 69 (45.4%) participants assumed that transfers occur during regular working hours from Monday to Friday, 8 a.m. to 4 p.m. There were significant percentage differences between the BRC and CMC groups (*p* = 0.037). Regular working hours were assumed by 64.3% of the BRC group and 34.4% of the CMC group. Instead, 25.0% of the CMC group assumed transfers during weekday on-call hours and 13.5% primarily on weekends. In total, 19.6% of the BMC group indicated the main transfer time during weekday duty hours and 5.4% on weekends. Furthermore, participants entered into the free text field that the emergency room is more likely to be signed off than patients being transferred, or generally no patients are transferred.

The majority of participants indicated that 0–10 patients (65.1%) are transferred per month, followed by 10–20 (12.5%) transfers. Most transfers take on average 1–6 h (46.7%), followed by a transfer duration of >24 h (15.8%). When asked about the transferring specialty, the majority of participants indicated trauma patients (63.2%), 10.5% were orthopedic transfers, and 9.2% indicated other reasons for patient transfers. For example, participants indicated that burn victims or patients with a leading neurosurgical diagnosis were transferred regularly.

The participants most frequently cited a lack of intensive care unit capacity as the cause of patient transfers (48.8%). A higher proportion was observed in the BRC group (48.2%) than in the CMC group (37.5%, *p* = 1). The second-most common reason was a lack of operating room capacity (35.5%). This was significantly more common in the CMC group (43.8%) than in the BRC group (21.4%, *p* = 0.007). Another relevant reason was the absence of specialist departments within the hospital (28.3%). This was significantly more common in the BRC group (50.0%) than in the CMC group (15.6%, *p* < 0.001). Additional reasons were a lack of approval for the SAV procedure (27.0%), staff shortage (19.1%), and lack of specialization (17.8%).

The most common reasons for transfers in orthopedics were patients with musculoskeletal tumors (27.0%). These patients were more frequently transferred in the BRC group (37.5%) than in the CMC group (20.8%, *p* = 0.745). This was followed by patients with periprosthetic infections in the BRC group (28.6%) and in the CMC group (10.4%). The third-most common reason for orthopedic transfers were patients with spondylodiscitis in the BRC group (26.8%) with only a small percentage in the CMC group (4.2%).

**Figure 1:**
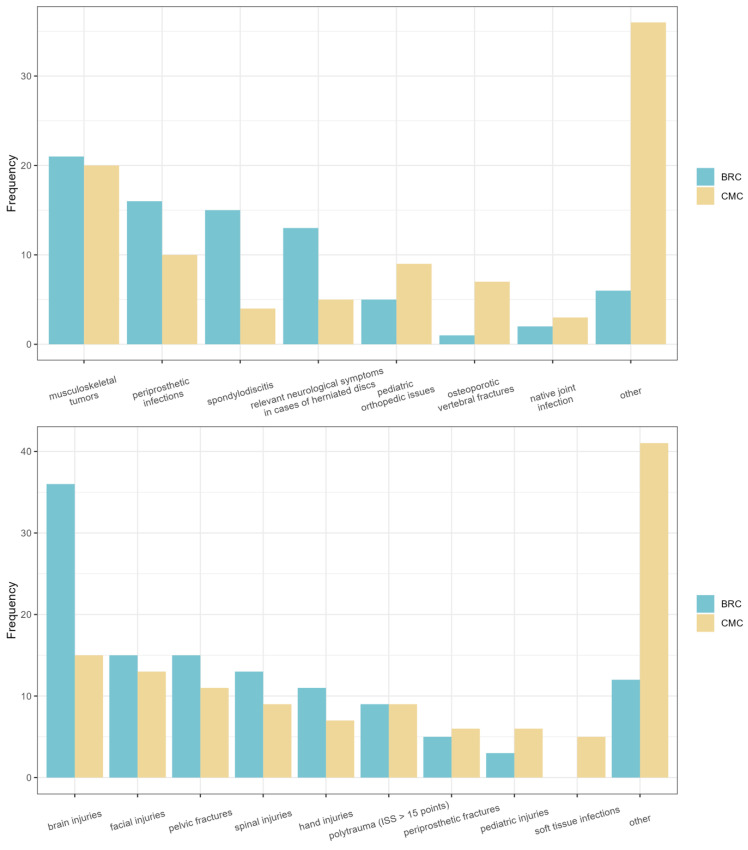
The figure displays the most common diagnoses for orthopedic and trauma surgery transfers for the BRC group and the CMC group. The upper chart represents the orthopedic transfers for both groups, while the lower chart depicts the trauma surgery transfers for both groups.

The most common reason for trauma patient transfers was traumatic brain injuries (33.6%). These were significantly more often transferred from the BRC group (64.3%) than from the CMC group (15.6%, *p* < 0.001). The second-most common were facial injuries (18.4%), and the third-most common injury pattern was pelvic fractures (17.1%). Facial injuries were more often transferred by the BRC group (26.8%) than by the CMC group (13.5%, *p* = 1). Other reasons for transfers included the SAV/VAV procedure due to lack of approval from the professional association or complex soft tissue defects. Polytrauma patients with an Injury Severity Score >15 only accounted for a small portion of the transfers (11.8%). An overview of the most common diagnoses is shown in Figure 1.

### 3.4. Communication and Quality

Forty-eight percent of the respondents indicated that they did not have a dedicated contact person in the receiving clinic. In particular, contact persons in the BRC group (55.4%) were more often missing than in the CMC group (43.8%, *p* = 1). The quality of patient transfers was rated as ‘good’ by the majority of participants (32.2%), followed by a ‘satisfactory’ rating (27.0%). Yet, the quality of communication was primarily rated as ‘satisfactory’ by the largest portion of participants (30.9%). A summary of the ratings for both groups, BRC and CMC, is presented in Table 4.

The most common form of communication was by far the telephone (82.2%). In addition, participants used teleradiology (25.0%), electronic image transmission (22.4%), and e-mail (13.2%). Fax (8.6%) and digital media (7.9%) were only used by a very small proportion of the participants.

The most common reasons for the delay in patient transfers were limited acceptance capacities (55.9%) or outstanding feedback from the receiving clinic (45.4%). Additional factors were waiting times for transport (36.2%), lack of accessibility of the receiving clinic (25.0%), and additional diagnostics desired before patient transfer (9.9%).

### 3.5. Proposals for Optimization

Participants were asked for suggestions for improvement in inter-clinical patient transfers. Suggestions included improvements in patient documentation with the option to submit subsequent findings (for example, microbiological examinations). Furthermore, the expansion of the use of digital media was suggested, as well as improving online communication and creating networks outside the existing ones.

Pre-clinically, it is recommended to optimize the distribution of emergency patients already by the emergency service and to improve the transferring structures. Especially, the duration of transport times was seen critically by the participants.

Further suggestions were central communication networks with the possibility of capacity queries, fixed contacts in the clinics, and a central bed overview for peripheral hospitals.

It is also recommended to adjust the reimbursement of transferred patients to the logistical extra effort and to develop standardized transfer requests as well as to create clear acceptance criteria and to improve digital data traffic.

An additional point of criticism and suggestion for optimization was the lack of accessibility and limited availability of decision makers and the possibility to contact them directly to avoid delays in transfers. Table 5 provides an overview of the submitted comments.

## 4. Discussion

The results presented from this survey provide an interesting insight into the structure and organization of hospital networks in orthopedics and trauma surgery. Overall, this study indicates that interhospital patient transfer is a common issue in orthopedics and trauma surgery that requires further optimization.

The comparison between large hospitals and smaller ones reveals that not only the smaller hospitals are affected by the problem. The reasons and treatment diagnoses differ both between BRC and CMC hospitals as well as within orthopedics and trauma surgery. The predominant reasons for transfers in orthopedics were patients with musculoskeletal tumors, accounting for 33.6% of all cases. These patients were transferred more frequently in the BRC group (37.5%) than in the CMC group (20.8%). At first glance, this proportion of transfers appears relatively high. Within the European context, it is worth noting that in the UK, there is a recommendation to refer every patient with a primary bone tumor to a specialized center for treatment by an accredited multidisciplinary bone sarcoma team [23]. Also, patients with primary malignant bone tumors appear to have a better overall survival in centers with a high patient volume than in facilities with a low patient volume [24]. The second-most common cause of transfers in the BRC group was patients with periprosthetic infections (28.6%), with a much lower percentage in the CMC group (10.4%). These infections necessitate complex treatment, can lead to extended hospital stays, and result in significant costs. The associated bone loss also presents challenges for the medical practitioner, and patients benefit from surgeons experienced in numerous revision procedures [25,26,27]. So this high proportion of transfers seems less surprising given the increasingly high proportion of revision arthroplasty procedures [28].

For trauma patient transfers, the leading cause was traumatic brain injuries, comprising 41.8% of all cases. These were significantly more often transferred from the BRC group (64.3%) than from the CMC group (15.6%). For this, the participants’ reported lack of monitoring beds is likely to be the main cause. Participants’ suggestions for improvement, such as a central bed overview or earlier patient selection in the ambulance service, could help reduce this proportion of transfers.

Also, a high proportion of transfers were reported for pelvic fractures. Here too, there seems to be an increase, particularly in geriatric fractures [29]. Pelvic fractures are associated with high mortality and morbidity and are challenging to treat [30,31]. In addition to the surgical challenge, the appropriate logistical conditions are also necessary to ensure care [32]. Intensive postoperative treatment is often required, which the participants frequently reported as lacking. Currently, there are no established treatment strategies for these patients, resulting in an increasing number of secondary treatments. In the UK, a guide for the treatment of pelvic fractures within the trauma network was introduced in 2018 to improve care [33]. In Germany, there currently are no care guidelines for this. An initiative to respond to the increasing number of geriatric patients is the introduction of the “AltersTraumaZentrum DGU^®^” to improve care [34]. With the ongoing hospital reform, the future development of the care situation is unclear. Regulations and guidelines for the treatment of complex disease patterns could certainly help to improve the care situation. So far, these do not exist in the care of orthopedic and trauma surgery patients, just as there are no clear recommendations and procedures on which patients should be transferred [15].

The significant difference in bed count between BRC and CMC hospitals suggests potential resource constraints, especially during peak times or emergencies. It is important to note that some participants provided a somewhat unrealistically high number of beds. In particular, the number of intensive care beds seems too high, with 465 in the BRC group and 200 in the CMC group. Interestingly, while BRC hospitals have a higher percentage of CTs available 24/7, the availability of MRIs in CMC hospitals is almost three times as high as in BRC hospitals. It is worth noting that a significant portion of the CMC group (24.0%) did not answer this question, which may give a false impression of higher CT availability in the BRC group. Certainly, a comprehensive overview of the availability of imaging diagnostics would be beneficial to prevent potential limitations in emergency care. Transfers during regular business hours may indicate a systemic issue. Rather than patient comfort or medical urgency, logistical or administrative factors might predominantly influence the decision to transfer. The main reasons for transfers, such as a lack of capacity in intensive care units or operating rooms, signal structural and resource-related challenges. These challenges seem more pronounced in BRC hospitals, suggesting disparities in resource allocation or hospital planning. The significant difference in the transfer of trauma patients between BRC and CMC, especially those with traumatic brain injuries, points to potential expertise or facility limitations in BRC hospitals.

The study encompasses all hierarchical positions in hospitals, with both chief physicians and assistant physicians equally represented. This diverse representation ensures comprehensive insights from all levels of management and expertise. Most participants come from maximum care hospitals located in large cities, making these results particularly relevant for urban, high-capacity settings. Interestingly, a high proportion of the survey participants are chief physicians and senior physicians. Yet, patient transfers are typically carried out by assistant physicians who have firsthand experience with the challenges of these transfers. The participants critically noted the lack of availability of senior physicians during on-call duty as a potential obstacle to timely and regulated patient transfers. The vast majority of hospitals are located in large cities. Therefore, the specific challenges and needs of hospitals in rural areas or small towns may be underrepresented in the study. Specific investigations are necessary to detect differences in patient transfers in cities and rural areas.

It is worth mentioning that in this study, it remains unclear which of the stated transfers are accepted and which patients are actually transferred. This should be further specified in future studies. Therefore, it remains uncertain what issues arise when admitting or transferring patients. Especially, it can be assumed that a larger portion of patients in the CMC group is accepted and not transferred. Participants indicate that they tend to transfer patients with minor injuries to smaller hospitals or need to transfer patients after surgical treatment, suggesting a lack of regular-ward bed capacity.

Some of the collected data, particularly those related to the quality of communication and assessment of patient transfers, are based on the subjective evaluations of the respondents. These evaluations could have been influenced by a variety of factors, including personal biases. The suggestions for optimization are interesting and could be useful in improving the processes of inter-clinical patient transfer. So far, the scientific basis for these evaluations is limited and requires further investigation, taking into account the mentioned aspects.

International comparisons also highlight the need for improvements in communication and process optimization [18]. Overall, the literature on transfers in orthopedics remains quite limited. Existing studies primarily focus on the appropriateness of transfers and the transfer of critically ill patients [16]. However, with the increasing number of periprosthetic infections and complications, standardized protocols are also necessary in this area and should be further researched [35].

Hospitals, especially those in the BRC category, might see advantages from targeted investments in specific specialties or facilities, considering the evident reasons for patient transfers. Establishing designated points of contact for transfers can streamline the process and elevate communication standards. Ongoing training and development can help in minimizing transfers of cases like traumatic brain injuries from BRC hospitals, enhancing accessibility of care for patients.

This study has several limitations. With a total of 152 participants, the sample size is relatively small, particularly considering the diversity of hospital types and positions represented in the study. Hence, the results might not be representative of all hospitals or medical positions. Additionally, there appears to be a certain imbalance in the distribution of participants between the BRC and CMC groups, which could impact the findings. For a more comprehensive analysis and also cross-disciplinary analysis in the future, it would be useful to collaborate with governmental bodies. This could help to create a broader and possibly more representative database, allowing a more reliable interpretation of the results in relation to the entire target group.

In conclusion, this study underscores the critical importance of developing enhanced protocols and allocating resources more effectively to address disparities in hospital transfers within the fields of orthopedics and trauma surgery in Germany.

## Figures and Tables

**Table 1:** Characteristics of participants stratified by hospitals of basic/regular care (BRC) and hospitals of central/maximum care (CMC).

		BRC (*n* = 56)	CMC (*n* = 96)	Total
Chief physicians	N (%)	17 (30.4%)	17 (17.7%)	34 (22.4%)
Senior doctors in a leading position	N (%)	13 (23.2%))	21 (21.9%)	34 (22.4%)
Senior doctors	N (%)	13 (23.2%)	27 (28.1%)	40 (26.3%)
Specialists	N (%)	2 (3.6%)	10 (10.4%)	12 (7.9%)
Residents	N (%)	11 (19.6%)	21 (21.9%)	32 (21.1%)
Rural community	N (%)	4 (7.1%)	1 (1.0%)	5 (3.3%)
Small town	N (%)	10 (17.9%)	3 (3.1%)	13 (8.6%)
Medium-sized city	N (%)	21 (37.5%)	23 (24.0%)	44 (28.9%)
Large city	N (%)	21 (37.5%)	69 (71.9%)	90 (59.2%)

**Table 2:** Comparison of hospitals of basic/regular care (BRC) and hospitals of central/maximum care (CMC) regarding size and available imaging capabilities. Group differences were assessed by Fisher’s exact test and Wilcoxon–Mann–Whitney U Test. CI: confidence interval for median (continuous variables) and percentages (for categorical variables).

		BRC	CMC	Total	*p*-Value
Beds	Median	50.0	78.0	60.0	<0.001
	[Min, Max]	[5.00, 465]	[5.00, 1000]	[5.00, 1000]	
	95% CI	(40, 50)	(64, 80)	(56, 70)	
Intensive care beds	Median	12.0	30.0	20.0	<0.001
	[Min, Max]	[3.00, 465]	[0, 200]	[0, 465]	
	95% CI	(11, 15)	(20, 38)	(15, 22)	
CT—Diagnostic	Yes				1.000
	N	48	71	119	
	(%, 95% CI)	(85.7%, 74–94)	(74.0%, 64–82)	(78.3%, 71–85)	
Missing answers		7 (12.5%)	23 (24.0%)		
MRI—Diagnostic	Yes				<0.001
	N	13	61	74	
	(%, 95% CI)	(23.2%, 13–36)	(63.5%, 53–73)	(48.7%, 41–57)	
Missing answers		7 (12.5%)	23 (24.0%)		

**Table 3:** Comparison of care structures and resources between basic/regular care (BRC) and hospitals of central/maximum care (CMC). CI: confidence interval for median (continuous variables) and percentages (for categorical variables).

		BRC	CMC	Total	*p*-Value
Number of specializations	Median	2	4	4	<0.001
	[Min, Max]	[1, 9]	[1, 13]	[1, 13]	
	95% CI	(1, 2)	(4, 5)	(3, 4)	
Hand surgery	N	22	77	99	<0.001
	(%, 95% CI)	(39.3%, 26–53)	(80.2%, 71–88)	(65.1%, 57–73)	
Severe injury procedure	N	7	70	77	<0.001
	(%, 95% CI)	(12.5%, 5–24)	(72.9%, 63–81)	(50.7%, 42–59)	
EndoProthetikZentrum (EPZ)	N	25	41	66	1.000
	(%, 95% CI)	(44.6%, 31–59)	(42.7%, 33–53)	(43.4%, 35–52)	
EndoProthetikZentrum of maximum care (EPZMAX)	N	6	33	39	0.016
	(%, 95% CI)	(10.7%, 4–22)	(34.4%, 25–45)	(25.7%, 19–33)	
Spine center of maximum care of the DWG	N	1	41	42	<0.001
	(%, 95% CI)	(1.8%, 0–10)	(42.7%, 33–53)	(27.6%, 21–35)	
Spine specialist center of the DWG	N	12	9	21	0.763
	(%, 95% CI)	(21.4%, 12–34)	(9.4%, 4–17)	(13.8%, 9–20)	
Spine facility of the DWG	N	15	13	28	0.778
	(%, 95% CI)	(26.8%, 16–40)	(13.5%, 7–22)	(18.4%, 13–26)	
Center for foot surgery	N	12	22	34	1.000
	(%, 95% CI)	(21.4%, 12–34)	(22.9%, 15–33)	(22.4%, 16–30)	
Rheumatological orthopedics	N	1	8	9	1.000
	(%, 95% CI)	(1.8%, 0–10)	(8.3%, 4–16)	(5.9%, 3–11)	
Hemophilia center	N	1	13	14	0.271
	(%, 95% CI)	(1.8%, 0–10)	(13.5%, 7–22)	(9.2%, 5–15)	
Tumor orthopedics	N	5	46	51	<0.001
	(%, 95% CI)	(8.9%, 3–20)	(47.9%, 38–58)	(33.6%, 26–42)	
Pediatric- and neuro-orthopedics	N	1	37	38	<0.001
	(%, 95% CI)	(1.8%, 0–10)	(38.5%, 29–49)	(25%, 18–33)	
Plastic reconstructive surgery	N	13	51	64	0.005
	(%, 95% CI)	(23.2%, 13–36)	(53.1%, 43–63)	(42.1%, 34–50)	
Other	N	8	9	17	1.000
	(%, 95% CI)	(14.8%, 6–26)	(9.4%, 4–17)	(11.3%, 7–17)	

**Table 4:** Comparison of communication quality and contact person availability between basic/regular care (BRC) and hospitals of central/maximum care (CMC). CI: confidence interval for percentages.

		BRC	CMC	Total	*p*-Value
Contact person		33.9% (CI: 22–48)	32.3% (CI: 23–43)	32.9% (CI: 25–41)	1.000
Quality of communication	Very good	8.9%	7.3%	7.9%	1.000
	Good	25.0%	28.1%	27.0%	
	Satisfactory	33.9%	29.2%	30.9%	
	Sufficient	14.3%	10.4%	11.8%	
	Insufficient	7.1%	5.2%	5.9%	

**Table 5:** The table displays the improvement suggestions provided in the comments. On the left are the comments from participants in the BRC group, and on the right are those from the CMC group.

BRC	CMC
For cases of limited admission capacities (especially in intensive care), assistance in bed searches.	From the perspective of the receiving clinic: Transferring hospitals often withhold crucial information that could jeopardize the possible acceptance of the patient, for example, mandatory isolation due to infectious diseases, misrepresentation of the urgency for transfer, lack of patient consent to possible therapies, exaggeration of clinical symptoms.
Improved accessibility of senior physicians.	Improved patient documentation. Opportunity for subsequent transmission of findings (e.g., Mibi).
Better feedback and increased willingness to accept.	Improved telemedicine/digital networking between hospitals.
Direct contact with a senior physician/decision-maker.	Database displaying available beds.
A single phone number with 24/7 availability with a knowledgeable and authorized contact person. If a conversation is currently being held, an automatic callback should be triggered.	Direct contact/availability of a decision maker.
A central phone number/contact person (on-duty senior physician) who is easier to reach.	Dedicated contacts, insufficient willingness to accept web-based image transfer (“better send a CD…”).
A designated contact person for transfers in each specialty; urgent training for the reception staff is necessary.	Release of an electronic file. A common data standard is required for this.
Dedicated contact persons, increased emergency capacity of maximum care providers.	Capacities, especially for emergencies during the night, should be distributed within the network.
Fixed and known uniform phone number for transfers; Clear acceptance criteria.	Clear levels of care.
Better management of transport capacities.	Clarification of possible readmissions.
Standardized transfer request.	Optimization of the distribution of emergency patients already by the emergency services.
Further possibilities to improve digital data traffic.	Specific phone numbers dedicated solely to the topic of patient transfers. Comprehensive teleradiology, use of data protection compliant modern communication media. Mutual transferability. Better compensation for patients who have been accepted to finance/compensate for the increased logistical effort.
Central communication network with the ability to query capacities.	Central “bed exchange” for specialty-specific peripheral hospitals.

## Data Availability

The datasets generated during and analyzed during the current study are available from the corresponding author upon reasonable request.

## Supplementary Materials

The following supporting information can be downloaded at: https://www.mdpi.com/article/10.3390/clinpract14030063/s1, Questionnaire with possible answers.
