# Supplementary material for: Optimizing Interhospital Transfers in Orthopedics and Trauma Surgery: Challenges, Insights, and Proposals for Standardized Care in Germany
Source: Clin Pract. 2024 May 8;14(3):789–800. doi: 10.3390/clinpract14030063 (PMC11130787; doi:10.3390/clinpract14030063)
Supplement: Supplementary file 1 [file clinpract-14-00063-s001.zip › clinpract-2884427-supplementary.pdf]

Variablen-Übersicht

Fragebogen-Interne Daten

Im Datensatz finden Sie neben Ihren Fragen folgende zusätzliche Variablen, sofern Sie die entsprechende Option beim Herunterladen des Datensatzes nicht deaktivieren.

CASE Fortlaufende Nummer der Versuchsperson
REF Referenz, falls solch eine im Link zum Fragebogen übergeben wurde
LASTPAGE Nummer der Seite im Fragebogen, die zuletzt bearbeitet und abgeschickt wurde
QUESTNNR Kennung des Fragebogens, der bearbeitet wurde
MODE Information, ob der Fragebogen im Pretest oder durch einen Projektmitarbeiter gestartet wurde
STARTED Zeitpunkt, zu dem der Teilnehmer den Fragebogen aufgerufen hat
FINISHED Information, ob der Fragebogen bis zur letzten Seite ausgefüllt wurde
TIME_001... Zeit, die ein Teilnehmer auf einer Fragebogen-Seite verbracht hat

Bitte beachten Sie, dass Sie die Fragebogen-internen Variablen nicht mit der Funktion value() auslesen können. Für Interview-Nummer und Referenz stehen aber die PHP-Funktionen PHP-Funktion caseNumber() und PHP-Funktion reference() zur Verfügung.

Details über die zusätzlichen Variablen stehen in der Anleitung: Zusätzliche Variablen in der Datenausgabe

Rubrik OU: Fragebogen

[OU01] Horizontale Auswahl
Position in der Klinik
"Was ist Ihre Position in der Klinik?"

OU01 Position in der Klinik
1 = Chef*ärztin
2 = Oberarzt*ärztin in leitender Funktion
3 = Oberarzt*ärztin
4 = Facharzt*ärztin
5 = Assistenzarzt*ärztin
-9 = nicht beantwortet

[OU02] Horizontale Auswahl
Versorgungsstufe
"Welche Versorgungsstufe hat Ihr Krankenhaus?"

OU02 Versorgungsstufe
1 = Grundversorgung (200 bis 299 Betten)
2 = Regelversorgung (300 bis 499 Betten)
3 = Zentralversorgung (500 bis 699 Betten)
4 = Maximalversorgung (700 bis über 1000 Betten)
5 = Sonstige (bitte angeben)
-9 = nicht beantwortet

OU02_05 Sonstige (bitte angeben)
Offene Texteingabe

[OU09] Horizontale Auswahl
Ortschaft
"Wo befindet sich Ihre Klinik?"

OU09 Ortschaft
1 = Landgemeinde (bis 5.000 Einwohner)
2 = Kleinstadt (bis 20.000 Einwohner)
3 = Mittelstadt (bis 100.000 Einwohner)
4 = Großstadt (ab 100.000 Einwohner)
5 = Sonstige (bitte angeben)
-9 = nicht beantwortet

OU09_05 Sonstige (bitte angeben)
Offene Texteingabe

[OU03] Horizontale Auswahl
Traumazentrum
"Ist Ihre Klinik als Traumazentrum zertifiziert?"

OU03 Traumazentrum
1 = Lokales Traumazentrum
2 = Regionales Traumazentrum
3 = Überregionales Traumazentrum
4 = Sonstiges (bitte angeben)
-9 = nicht beantwortet

OU03_04 Sonstiges (bitte angeben)
Offene Texteingabe

[OU04] Texteingabe mit Auswahlempfehlung
Betten
"Wieviele Betten hat Ihre Abteilung?"

OU04 Betten
-2 = sonstige Texteingabe
-9 = nicht beantwortet

OU04s Betten (offene Eingabe)
Offene Texteingabe

[OU05] Texteingabe mit Auswahlempfehlung
Intensivbetten
"Über wieviele Intensivbetten verfügt Ihre Klinik?"

OU05 Intensivbetten
-2 = sonstige Texteingabe
-9 = nicht beantwortet

OU05s Intensivbetten (offene Eingabe)
Offene Texteingabe

[OU06] Mehrfachauswahl
Spezialisierung
"Hat Ihre Abteilung eine oder mehrere der folgenden Spezialisierungen?"

OU06 Spezialisierung: Ausweichoption (negativ) oder Anzahl ausgewählter Optionen
Ganze Zahl
OU06_01 Handchirurgie
OU06_02 SAV Verfahren
OU06_03 EndoProthetikZentrum (EPZ)
OU06_04 Endoprothesenzentrum der Maximalversorgung (EPZMAX)
OU06_05 Wirbelsäulenzentrum der Maximalversorgung der DWG®
OU06_06 Wirbelsäulenspezialzentrum der DWG®
OU06_07 Wirbelsäuleneinrichtung der DWG®
OU06_08 Fußzentrum
OU06_09 Rheumaorthopädie
OU06_10 Haemophilie
OU06_11 Tumororthopädie
OU06_12 Kinder-und Neuroorthopädie
OU06_13 Plastisch-rekonstruktive Chirurgie
OU06_14 Sonstige (bitte angeben)
1 = nicht gewählt
2 = ausgewählt
OU06_14a Sonstige (bitte angeben) (offene Eingabe)
Offene Texteingabe

[OU07] Mehrfachauswahl
Gründe
"Was sind die häufigsten Gründe für Ihre Verlegungen?"

OU07 Gründe: Ausweichoption (negativ) oder Anzahl ausgewählter Optionen
Ganze Zahl
OU07_01 Personalmangel
OU07_02 Fehlende Intensivkapazitäten
OU07_03 Kritische Kostendeckung
OU07_04 Fehlende OP-Kapazitäten
OU07_05 Mangelnde Spezialisierung
OU07_06 Fehlende Fachabteilungen (Neurochirurgie, Augenheilkunde etc.)
OU07_07 Keine ausreichende Zulassung (SAV Verfahren)
OU07_08 Sonstige (bitte angeben)
1 = nicht gewählt
2 = ausgewählt
OU07_08a Sonstige (bitte angeben) (offene Eingabe)
Offene Texteingabe

[OU08] Texteingabe mit Auswahlempfehlung
Anzahl
"Wieviele Patienten müssen ca. monatlich bei Ihnen verlegt werden?"

OU08 Anzahl
-2 = sonstige Texteingabe
-9 = nicht beantwortet
OU08s Anzahl (offene Eingabe)
Offene Texteingabe

[OU10] Horizontale Auswahl
Zeitpunkt
"Zu welchem Zeitpunkt finden Ihrer Meinung nach die meisten Verlegungen statt?"

OU10 Zeitpunkt
1 = Regelarbeitszeit (Montag - Freitag von 8 bis 16 Uhr)
2 = Dienstzeit (Montag bis Freitag von 16 bis 8 Uhr)
3 = Wochenende (Freitag bis Montag von 16 bis 8 Uhr)
4 = Sonstige (bitte angeben)
-9 = nicht beantwortet

OU10_04 Sonstige (bitte angeben)
Offene Texteingabe

[OU11] Horizontale Auswahl
Ansprechpartner
"Haben Sie für Ihre Verlegungen einen festen Ansprechpartner?"

OU11 Ansprechpartner
1 = Ja
2 = Nein
3 = Sonstige (bitte angeben)
-9 = nicht beantwortet

OU11_03 Sonstige (bitte angeben)
Offene Texteingabe

[OU12] Mehrfachauswahl
Kommunikation
"Wie kommunizieren Sie mit dem anderen Krankenhaus?"

OU12 Kommunikation: Ausweichoption (negativ) oder Anzahl ausgewählter Optionen
Ganze Zahl

OU12_01 Telefon

OU12_02 Fax

OU12_03 Mail

OU12_04 Digitale Medien

OU12_05 Elektrische Bildübertragung

OU12_07 Teleradiologie

OU12_06 Sonstige (bitte angeben)
1 = nicht gewählt
2 = ausgewählt

OU12_06a Sonstige (bitte angeben) (offene Eingabe)
Offene Texteingabe

[OU18] Horizontale Auswahl
Komunikationsqualität
"Wie würden Sie die Kommunikation bei Verlegeanfragen bewerten?"

OU18 Komunikationsqualität
1 = Sehr gut
2 = Gut
3 = Befriedigend
4 = Ausreichend
5 = Ungenügend
6 = Sonstige (bitte angeben)
-9 = nicht beantwortet

OU18_06 Sonstige (bitte angeben)
Offene Texteingabe

[OU19] Horizontale Auswahl
Dauer
"Wie lange dauert es im Schnitt Ihrer Meinung nach von der ersten Anfrage bis zur definitiven Verlegung?"

OU19 Dauer
1 = < 1 Stunde
2 = 1 bis 6 Stunden
3 = > 6 Stunden
4 = > 12 Stunden
5 = > 24 Stunden
6 = Sonstige (bitte angeben)
-9 = nicht beantwortet

OU19_06 Sonstige (bitte angeben)
Offene Texteingabe

[OU20] Horizontale Auswahl
Notfalldiangostik CT
"Verfügen Sie über die Möglichkeit einer 24/7 CT-Diagnostik?"

OU20 Notfalldiangostik CT
1 = Ja
2 = Nein
3 = Sonstige (bitte angeben)
-9 = nicht beantwortet

OU20_03 Sonstige (bitte angeben)
Offene Texteingabe

[OU13] Horizontale Auswahl
Fachrichtung
"Verlegen Sie mehr orthopädische oder unfallchirurgische Patienten?"

OU13 Fachrichtung
1 = Orthopädie
2 = Unfallchirurgie
3 = Sonstige (bitte angeben)
-9 = nicht beantwortet

OU13_03 Sonstige (bitte angeben)
Offene Texteingabe

[OU14] Mehrfachauswahl
Diagnosen O
"Was sind die häufigsten Diagnosen für Ihre orthopädischen Verlegungen?"

OU14 Diagnosen O: Ausweichoption (negativ) oder Anzahl ausgewählter Optionen
Ganze Zahl

OU14_01 Periprothetischer Infekt
OU14_02 Nativer Gelenkinfekt
OU14_03 Osteoporotische Wirbelkörperfrakturen
OU14_04 Muskuloskelettale Tumor
OU14_05 Kinderorthopädische Fragestellungen
OU14_06 Spondylodiszitis
OU14_07 Relevante neurologische Symptomatiken bei Bandscheibenvorfallen
OU14_08 Sonstige (bitte angeben)
1 = nicht gewählt
2 = ausgewählt

OU14_08a Sonstige (bitte angeben) (offene Eingabe)
Offene Texteingabe

[OU15] Mehrfachauswahl
Diagnosen U
"Was sind die häufigsten Diagnosen für Ihre unfallchirurgischen Verlegungen?"

OU15 Diagnosen U: Ausweichoption (negativ) oder Anzahl ausgewählter Optionen
Ganze Zahl
OU15_01 Handverletzungen
OU15_02 Periprothetische Frakturen
OU15_03 Beckenfrakturen
OU15_04 Wirbelsäulenverletzungen
OU15_05 Weichteilinfekte
OU15_06 Schädel-Hirn-Traumata
OU15_07 Gesichtverletzungen
OU15_08 Kindliche Verletzungen
OU15_10 Polytrauma (ISS > 15 Punkte)
OU15_09 Sonstige (bitte angeben)
1 = nicht gewählt
2 = ausgewählt
OU15_09a Sonstige (bitte angeben) (offene Eingabe)
Offene Texteingabe

[OU16] Horizontale Auswahl
Qualität
"Wie würden Sie die Erreichbarkeit bei Verlegeanfragen bewerten?"

OU16 Qualität
1 = Sehr gut
2 = Gut
3 = Befriedigend
4 = Ausreichend
5 = Ungenügend
6 = Sonstige (bitte angeben)
-9 = nicht beantwortet
OU16_06 Sonstige (bitte angeben)
Offene Texteingabe

[OU17] Texteingabe mit Auswahlempfehlung
Optimierung
"Welche Optimierungsmöglichkeiten sehen Sie bei den Patientenverlegungen?"

OU17 Optimierung
-2 = sonstige Texteingabe
-9 = nicht beantwortet
OU17s Optimierung (offene Eingabe)
Offene Texteingabe

[OU21] Horizontale Auswahl
Notfalldiangostik MRT
"Verfügen Sie über die Möglichkeit einer 24/7 MRT-Diagnostik?"

OU21 Notfalldiangostik MRT
1 = Ja
2 = Nein
3 = Sonstige (bitte angeben)
-9 = nicht beantwortet
OU21_03 Sonstige (bitte angeben)
Offene Texteingabe

[OU22] Mehrfachauswahl
Verzögerungen
"Was ist Ihrer Meinung nach der Hauptgrund für Verzögerungen bei den Verlegungen?"

OU22 Verzögerungen: Ausweichoption (negativ) oder Anzahl ausgewählter Optionen
Ganze Zahl

OU22_01 Mangelnde Erreichbarkeit der Empfängerklinik
OU22_02 Wartezeiten für den Patiententransport
OU22_03 Ausstehende Rückmeldungen der Empfängerklinik
OU22_04 Eingeschränkte Übernahmekapazitäten
OU22_05 Ergänzende Diagnostik vor der Übernahme
OU22_06 Sonstige (bitte angeben)
1 = nicht gewählt
2 = ausgewählt

OU22_06a Sonstige (bitte angeben) (offene Eingabe)
Offene Texteingabe
